# Distinct patterns of notochord mineralization in zebrafish coincide with the localization of Osteocalcin isoform 1 during early vertebral centra formation

**DOI:** 10.1186/1471-213X-12-28

**Published:** 2012-10-09

**Authors:** Anabela Bensimon-Brito, João Cardeira, Maria Leonor Cancela, Ann Huysseune, Paul Eckhard Witten

**Affiliations:** 1 PhD Program in Biomedical Sciences, University of Algarve, Faro, Portugal; 2 Center of Marine Sciences - CCMar, University of Algarve, Faro, Portugal; 3 Evolutionary Developmental Biology, Biology Department, Ghent University, Ghent, Belgium; 4 Dept. of Biomedical Sciences and Medicine, University of Algarve, Faro, Portugal; 5 Present address: CEDOC - Faculdade de Ciências Médicas, FCM Universidade Nova de Lisboa, Lisbon, Portugal

**Keywords:** Vertebral column, Vertebral fusion, Notochord, Osteocalcin

## Abstract

**Background:**

In chondrichthyans, basal osteichthyans and tetrapods, vertebral bodies have cartilaginous anlagen that subsequently mineralize (chondrichthyans) or ossify (osteichthyans). Chondrocytes that form the vertebral centra derive from somites. In teleost fish, vertebral centrum formation starts in the absence of cartilage, through direct mineralization of the notochord sheath. In a second step, the notochord is surrounded by somite-derived intramembranous bone. In several small teleost species, including zebrafish (*Danio rerio*), even haemal and neural arches form directly as intramembranous bone and only modified caudalmost arches remain cartilaginous. This study compares initial patterns of mineralization in different regions of the vertebral column in zebrafish. We ask if the absence or presence of cartilaginous arches influences the pattern of notochord sheath mineralization.

**Results:**

To reveal which cells are involved in mineralization of the notochord sheath we identify proliferating cells, we trace mineralization on the histological level and we analyze cell ultrastructure by TEM. Moreover, we localize proteins and genes that are typically expressed by skeletogenic cells such as Collagen type II, Alkaline phosphatase (ALP) and Osteocalcin (Oc). Mineralization of abdominal and caudal vertebrae starts with a complete ring within the notochord sheath and prior to the formation of the bony arches. In contrast, notochord mineralization of caudal fin centra starts with a broad ventral mineral deposition, associated with the bases of the modified cartilaginous arches. Similar, arch-related, patterns of mineralization occur in teleosts that maintain cartilaginous arches throughout the spine.

Throughout the entire vertebral column, we were able to co-localize ALP-positive signal with chordacentrum mineralization sites, as well as Collagen II and Oc protein accumulation in the mineralizing notochord sheath. In the caudal fin region, ALP and Oc signals were clearly produced both by the notochord epithelium and cells outside the notochord, the cartilaginous arches. Based on immunostaining, real time PCR and *oc2:gfp* transgenic fish, we identify Oc in the mineralizing notochord sheath as osteocalcin isoform 1 (Oc1).

**Conclusions:**

If notochord mineralization occurs prior to arch formation, mineralization of the notochord sheath is ring-shaped. If notochord mineralization occurs after cartilaginous arch formation, mineralization of the notochord sheath starts at the insertion point of the arches, with a basiventral origin. The presence of ALP and Oc1, not only in cells outside the notochord, but also in the notochord epithelium, suggests an active role of the notochord in the mineralization process. The same may apply to Col II-positive chondrocytes of the caudalmost haemal arches that show ALP activity and Oc1 accumulation, since these chondrocytes do not mineralize their own cartilage matrix. Even without cartilaginous preformed vertebral centra, the cartilaginous arches may have an inductive role in vertebral centrum formation, possibly contributing to the distinct mineralization patterns of zebrafish vertebral column and caudal fin vertebral fusion.

## Background

The structural units of the vertebral column are the vertebrae, composed of neural and haemal arches and the vertebral body itself, the centrum. Vertebral bodies are joined by intervertebral tissue primarily derived from the notochord [1-3].

The vertebral column results from a strictly controlled segmentation process that occurs in all vertebrate species and is associated to two main structures, the notochord and the somites [4,5]. The somites are epithelialized spheres of mesoderm that develop on either side of the neural tube, give rise to dermis, skeletal musculature (dermomyotome) and vertebrae (sclerotome) [6,7]. While the somite contribution to vertebra formation has been extensively studied, particularly in birds [6,8], the role of the notochord has received less attention. In teleosts, the majority of extant vertebrates, the notochord is composed of a core of large, vacuolated chordocytes, and an epithelial layer of chordoblasts that secrete the notochord sheath [9]. The notochord sheath is a stratified structure, composed of a thin external membrane, with high elastin content, covering a thicker collagenous layer [10,11].

In chondrichthyans and most osteichthyans, including tetrapods, but not in teleosts, vertebral bodies have a cartilaginous anlage that subsequently either mineralizes or is replaced by bone. In teleosts such as zebrafish, vertebral centra are formed in the absence of cartilage [12,13]. Indeed, teleost vertebral centra form through the mineralization of the notochord sheath (chordacentrum), which is then surrounded by somite-derived intramembranous bone (autocentrum) [2,12,14-16].

In teleosts, such as Atlantic salmon (*Salmo salar*) or zebrafish (*Danio rerio*), the notochord plays an important role in early life stages, as its mechanical function is only replaced by the vertebral column in the postembryonic life [2,17,18]. In Atlantic salmon, the initial mineralization of the chordacentrum has been described to be associated with cells of the notochord epithelium (chordoblasts), while bone formation, by sclerotome-derived cells (autocentrum), is a second step [2]. In contrast, Inohaya and co-workers suggest that in medaka (*Oryzias latipes*), only sclerotome-derived cells are involved in chordacentrum and autocentrum mineralization, with no role of chordoblasts [19]. Yet, a recent study [20] shows that, also in medaka, with conditional ablation of osterix-positive osteoblasts, notochord sheath mineralization is maintained. Therefore, the main cellular and molecular determinants involved in early notochord mineralization are still under debate. Furthermore, the role of extracellular matrix proteins such as osteocalcin, generally expressed by mature and resting osteoblasts [21] and by hypertrophic chondrocytes [17,22-24], in that process, remains unclear. In several teleosts, including zebrafish, two *Osteocalcin* genes (*Oc1* and *Oc2*) have been identified [25].

In amniotes the vertebral column is divided into five main regions whereas the vertebral column of teleosts is often only subdivided into two main regions, abdominal and caudal (e.g., [26-28]). However, also in zebrafish, several regions can be recognized within the vertebral column. In particular, regions that contain the most anterior and the most posterior vertebrae are highly specialized [15,29]. Regional differences are not only apparent at the morphological level but also regarding the tendency of vertebrae to fuse. While zebrafish vertebral bodies usually display no pathological fusion [30], caudal fin vertebrae undergo several fusions as part of regular development [15,30-32]. Yet, other teleosts, such as Atlantic salmon and other farmed species, are known to suffer frequent pathological vertebral fusions [33,34]. Whether regional differences in vertebrae morphology and mineralization relate to the susceptibility to fuse remains an open question.

This study aims to characterize mineralization patterns in different regions of the vertebral column (abdominal, caudal, caudal fin region) using various methods to reveal mineral deposition. Subsequently, these patterns are compared with the histogenesis of the arches, revealed through Collagen type II immunostaining, and to the timing of centrum formation. We characterize the proliferation of notochord cells and we also localize proteins related to mineralization, such as Alkaline phosphatase and Osteocalcin. We here provide the first evidence for the early presence of Osteocalcin 1 in mineralizing chordacentra. Finally, we discuss a possible association between timing of centra formation, the mineralization pattern and occurrence of vertebral fusion.

## Results

### Centrum mineralization follows arch formation in the caudal fin region

**Figure 1:**
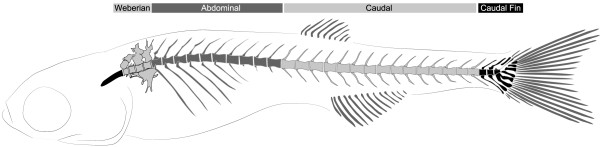
**Zebrafish vertebral column regions.** Weberian, abdominal, caudal and caudal fin regions are identified in different shades of grey.

The fully developed zebrafish vertebral column displays a mode of 33 vertebral bodies (Figure 1) including 4 centra in the Weberian apparatus, 10 abdominal vertebrae, 15 caudal vertebrae, and 3 to 4 caudal fin vertebrae (depending on the presence of a fourth preural) [15].

**Figure 2:**
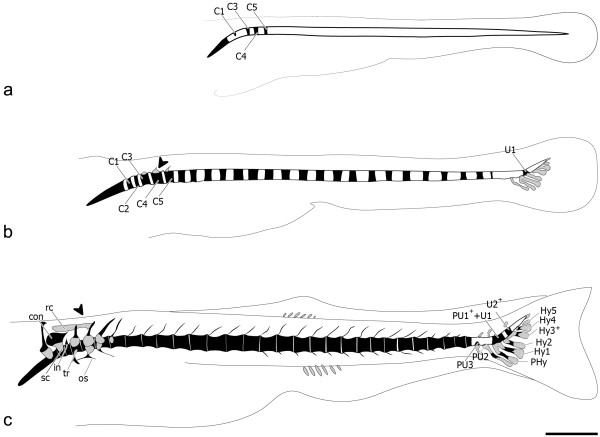
**Zebrafish vertebral column development.** Lateral view (**a**) of 4.4 mm TL zebrafish (*Danio rerio)* vertebral column. In black are the mineralized structures, demonstrating the formation of centrum 1 (C1) and the presence of C3 to C5. (**b**) At 5.5 mm TL caudal fin vertebrae formation with broad base origin can already be seen, represented by U1, and anterior vertebrae also develop, displaying a ring-shaped pattern of mineralization. In grey are the cartilaginous structures, including the caudal fin modified haemal arches and the anterior C3-C5 neural arches (black arrowhead). (**c**) At 6.0 mm TL PU3 appears, followed by the last vertebral body to form in the vertebral column, PU2, formed around 6.7 mm TL. At this stage, most anterior vertebral bodies already show bone formation around the notochord sheath. The Weberian apparatus is already well differentiated. Further abbreviations: C – centrum; con – concha scaphium; Hy1-5 – hypurals 1 to 5; in – intercalarium; os – os suspensorium; PHy – parhypural; PU1^+^+U1 – compound centrum preural 1 and ural 1; PU2-3 – preurals 2 and 3; rc – roofing cartilage; sc – scaphium; tr – tripus; U2^+^ – ural 2; (+) sign indicates vertebral elements that are the product of fusion events. Scale bar: 1 mm.

The first centra to mineralize (Figure 2a) are 3 and 4 (4.0 mm TL) in an anterior-to-posterior direction, followed by the mineralization of centrum 5, and later centra 1 and 2, in specimens of 4.4 mm TL. The mineralization of the remaining abdominal and caudal centra follows an anterior-to-posterior direction (Figure 2b). The mineralization of the caudal fin centra starts with the U1 from the compound centrum [PU1^+^+U1], in specimens of 5.5 mm TL (Figure 2b). PU2 is the last centrum to be formed, in specimens of 6.7 mm TL, preceded by PU3 formation (Figure 2c). By then, the complex cartilaginous structure that forms the Weberian apparatus is already present in the anterior part of the vertebral column.

**Figure 3:**
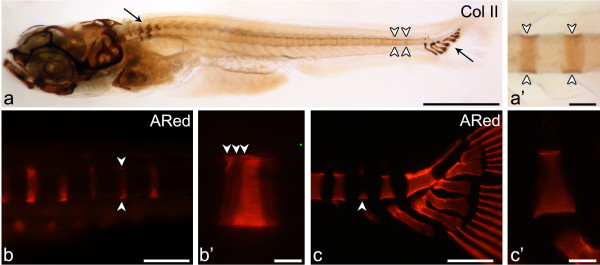
**Early mineralization stages of vertebral centra and association with cartilaginous arches.** Lateral view of (**a**, **a’**) Collagen type II immunostaining in the vertebral column of a 5.9 mm TL zebrafish shows (**a**) protein accumulation mostly in the cartilaginous structures of the Weberian apparatus and of the caudal fin skeleton (arrows) but also in the notochord sheath (white arrowheads). (**a’**) Although less evident than in cartilage, the staining can be clearly identified in the notochord matrix in a segmented manner (white arrowheads). (**b**-**c**) Early mineralization can be detected through alizarin red S (ARed) staining viewed under fluorescent light. (**b**) Early stage “ring” centrum (arrowheads) and (**b’**) close up showing the mineralization fronts (white arrowheads) in 5.0 mm TL fish. (**c**) Caudal fin centrum mineralization with a basiventral origin (arrowhead) and (**c’**) close up presenting a uniform mineralization surface with no distinct mineralization fronts, here represented by a 6.5 mm TL fish. Scale bars (**a**): 1 mm; (**a’**) 0.1 mm; (**b**,**c**): 0.15 mm; (**b’**, **c’**) 30 μm.

All Weberian, abdominal and caudal centra mineralize before development of the arches (Figure 3a). In the Weberian region, the haemal and neural arches develop as cartilaginous anlagen. These are Collagen type II positive, except for the associated spines that have no cartilaginous precursor. In the abdominal and caudal regions, the arches develop as intramembranous bones, with no cartilaginous anlage.

In contrast, in the modified caudal fin vertebrae, centrum mineralization occurs after arch formation. The (modified) arches associated to these centra have a cartilaginous anlage, also positive for Collagen type II. Staining of these arches was particularly strong at the bases (attachment areas to the notochord sheath) and distal parts, where no perichondral mineralization is present throughout the individual’s life span. Collagen type II immunostaining also showed protein accumulation in the notochord sheath, although less evident than in the cartilage (Figure 3a’).

In addition to the different timing of centrum mineralization versus arch formation, the present results also show a clear pattern concerning mineral expansion within the centra (Figure 3b, b’, c, c’).

With the exception of C1 and C2, vertebral bodies in the Weberian, abdominal and caudal regions start to mineralize in the form of a ring-shaped mineralized structure that expands in both anterior and posterior directions (Figure 3b and b’). These centra will be referred to as “ring centra”. In none of these vertebral bodies, have arches ever been observed prior to centrum mineralization. Nevertheless, the origin of mineralization matches the position of the myosepta, i.e. the site where neural and haemal arches will develop at a later stage. From this point, centrum mineralization expands one quarter anteriorly and three quarters posteriorly, showing clear incremental mineralization fronts (Figure 3b’).

Different from this pattern, in the caudal fin (PU4/3 to U2) region, mineralization starts as a broad ventral mineral deposition (basiventral origin) (Figure 3c and c’), which expands dorsally. Here, mineralization starts and expands from the attachment sites of the already developed modified cartilaginous haemal arches to the notochord. The developing centra show no apparent growth fronts as seen in the “ring centra” (Figure 3c’).

### Inner and outer cell distribution during chorda- and autocentrum formation

**Figure 4:**
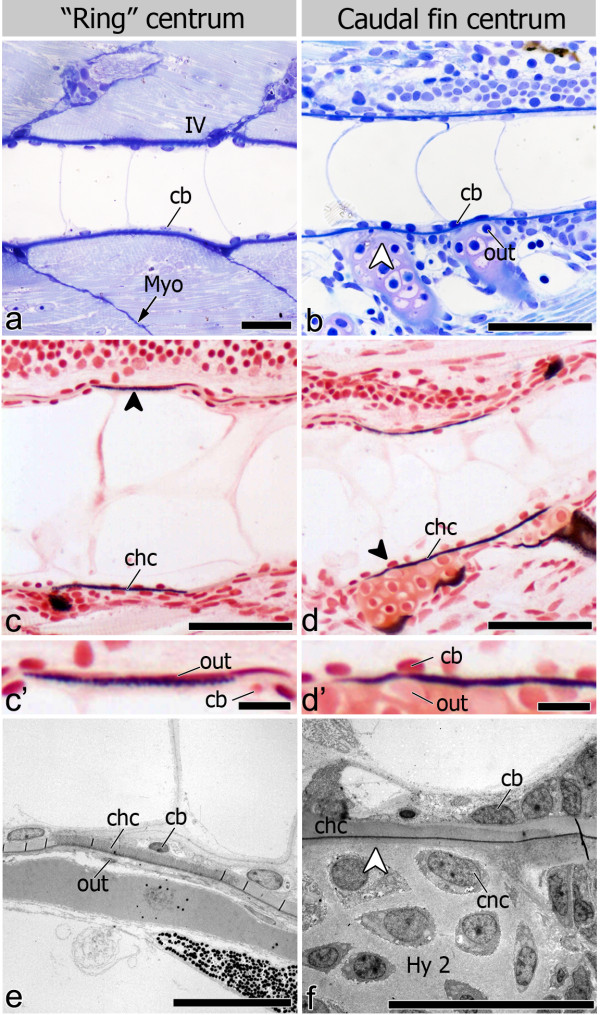
**Chordacentrum formation and cell distribution inside and outside the notochord.** Lateral view of 6.0 mm TL fish (**a**-**d’**). (**a**, **b**) Toluidine blue stained sections show the distinct morphologies of (**a**) “ring” and (**b**) caudal fin chordacentrum but homogenous chordoblast (cb) distribution during chordacentrum mineralization. In caudal fin vertebrae, outer (out) cells associated with the centra are chondrocytes from the cartilaginous arches (white arrowhead); (**c** and **d**) von Kossa staining highlighting that early (**c**) “ring” and (**d**) caudal fin centrum mineralization occurs within the notochord sheath, thus forming the chordacentrum (chc; black arrowheads); (**c’** and **d’**) details showing outer and inner cells in both (**c’**) “ring” (dorsal side) and (**d’**) caudal fin centra (ventral side). Lateral view of (**e**) 5 mm TL fish in TEM micrograph showing that both chordoblasts and outer cells (out) display a squamous morphology, associated with sites of “ring” chordacentrum mineralization, while in caudal fin centra (**f**) the cartilaginous matrix of the arches is directly attached to the notochord sheath (white arrowhead), leaving no space for cells other than chondrocytes (cnc) to interact with the chordacentrum outside the notochord (11 mm TL fish). Further abbreviation: Hy2 – Hypural 2; IV – intervertebral space; Myo – myoseptum. Scale bars (**a**-**d**): 50 μm; (**c’**, **d’**): 10 μm; (**e**, **f**): 20 μm.

**Figure 5:**
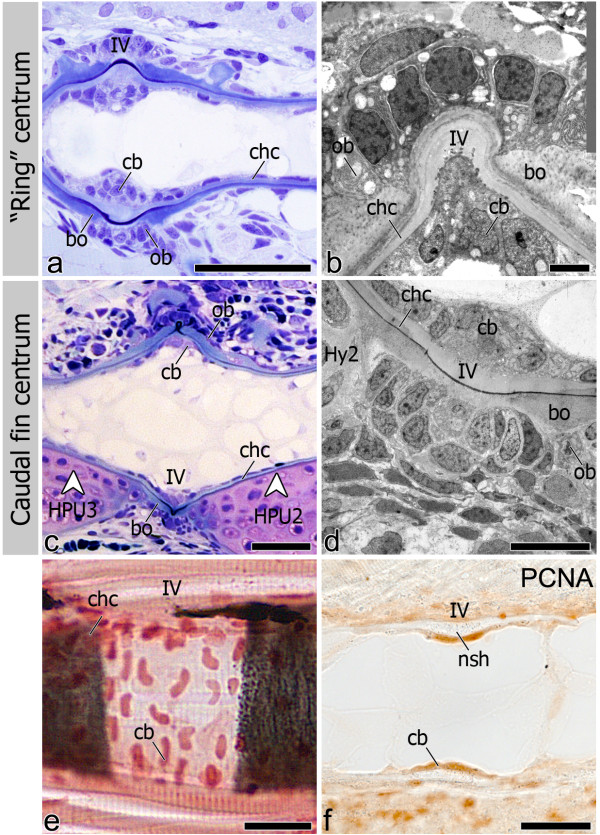
**utocentrum development and chordoblast positioning in the intervertebral space.** Lateral view of (**a**) Toluidine blue stained section shows bone (bo) deposition around the chordacentrum (chc), forming the autocentrum in “ring” vertebrae. (**b**) TEM micrograph showing the intervertebral region (IV). Osteoblasts (ob) are clearly associated with bone deposition. (**c**) Autocentrum formation also follows chordacentrum development in caudal fin vertebrae although with apparent less amount of bone and osteoblasts due to the presence of the cartilaginous arches (white arrowheads). (**d**) TEM micrograph of the intervertebral (IV) region between two caudal fin centra showing the bone associated osteoblasts. (**e**) Sagittal section through the notochord at the level of the IV space. Once chordacentra are fully defined, as shown by von Kossa staining, chordoblasts (cb) tend to accumulate in the intervertebral region, acquiring an oblong shape, larger size, and positioning in a dorsal-ventral direction. (**f**) Sagittal section through the notochord at the level of the IV space. At this stage chordoblasts become highly proliferative, as confirmed by PCNA positive staining. Sections from panels a-d are from 11 mm TL fish, while e-f represent an earlier developmental stage with 6 mm and 6.2 mm TL fish, respectively. Further abbreviations: HPU2-3 - Haemal arch of Preural 2 and 3; Hy2 – Hypural 2; nsh – notochord sheath. Scale bars (**a**, **c**): 50 μm; (b, d): 10 μm; (**e**, **f**): 30 μm.

Irrespective of the shape of the mineralized centrum, (“ring” and caudal fin centra) (Figure 4a, b), the origin of mineralization was shown both with von Kossa staining (Figure 4c, c’, d, d’) and TEM (Figure 4e-f), to occur always within the notochord sheath, thus establishing the chordacentrum. Once the chordacentrum is fully formed, perichordal bone is deposited mostly at the anterior and posterior edges, in this way establishing the vertebral endplates of the centra, thus forming the autocentrum (Figure 5a-d). Autocentrum formation occurs irrespective of the initial mineralization pattern within the notochord sheath (“ring” vs. basiventral origin). Yet, in the caudal fin centra, decreased bone formation is observed in the areas where cartilaginous arches attach to the notochord (Figure 5c-d).

During chordacentrum formation, notochord inner cells or chordoblasts do not accumulate in the prospective intervertebral space (Figure 4a, b), unlike what has been observed in other teleost species. However, once the centrum is mineralized, a higher number of chordoblasts can be seen in the non-mineralized intervertebral space (Figure 5a, c, e). By then, the chordoblasts are oblong, larger in size and are oriented in a dorsal-ventral direction (Figure 5e), as opposed to the squamous morphology displayed prior to and during chordacentrum development. In addition, chordoblasts proliferate in the intervertebral space, as shown by PCNA staining. Here, notochord sheath thickening occurs (Figure 5f). This pattern of chordoblast distribution is observed throughout the notochord, regardless of the type of centrum and its mineralization pattern (Figure 5a-d).

During chordacentrum formation, contrary to the inner notochord cells, the assumed sclerotome-derived notochord outer cells [35] in the “ring” vertebrae are visible as few scattered nuclei located around the notochord (Figure 4e). These cells are squamous and without apparent morphological features of active osteoblasts. In the caudal fin region, the cartilaginous haemal arches of the PUs (parhypural and hypurals 1, 2 and 4) directly attach to the notochord sheath with no space for other cells in-between the notochord sheath and the chondrocytes of the arches (Figure 4f).

During autocentrum formation, osteoblasts can be clearly identified in association with bone deposited around the “ring” chordacentra (Figure 5a). Major areas of the caudal fin chordacentra are covered by the cartilaginous arches attached to the notochord sheath surface (Figure 5c). Yet some osteoblasts can be identified wherever the arches are absent, lining the edges of the prospective vertebral endplates.

### Osteocalcin in chordacentrum formation

**Figure 6:**
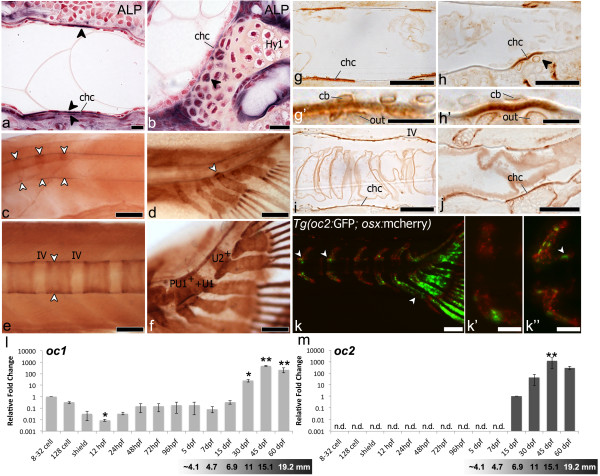
**Mineralization and centrum formation, lateral views, anterior left.** (**a**) ALP in a “ring”-centrum (5.0mm TL) adjacent to chordacentrum (chc) mineralization site (arrowheads). (**b**) In caudal fin centrum (6.2mm TL) ALP (arrowhead) in arch-chondrocytes next to chordacentrum. Fast red counterstaining. Osteocalcin (arrowheads) in early mineralized (**c**) “ring”-centra (4mm TL), and (**d**) caudal fin centra (5.9mm TL). Osteocalcin in later stages: (**e**) “Ring”-centra (5.9mm TL), incremental marks (arrowheads), and (**f**) caudal fin centra, uniform staining. Sections of wholemount Osteocalcin immunostaining (**g**-**j**). In early chordacentra (5.9mm TL) Osteocalcin accumulates in the notochord sheath in: (**g**) “ring”-centra and (h) caudal fin centra. Co-localization with arch attachment (arrowhead). (**g’**) Detail of (**g**). Chordoblasts (cb), outer cells (out). (**h’**) Detail of (**h**). Osteocalcin-positive chondrocytes (out). In advanced chordacentra (**i**, **j**) uniform Osteocalcin accumulation in the matrix, 6.1mm and 6.9mm TL. (**k**) Tg(oc2:gfp; osx:mcherry) transgenic fish, 12mm TL. oc2-positive cells (green), at neural and haemal arches next to “ring”-centra (arrowheads). Osx-positive cells (red) cover vertebral centra. Gradient between (**k’**) less developed (oc2-negative) and (**k”**) more developed (oc2-positive) centra. (**k”**) Oc2 cells (white-arrowhead) next to an autocentrum. (**l**, **m**) qPCR, relative gene expression. oc1 and oc2. First detection of transcripts is the reference, represented by a 1-fold change (oc1:8-32 cells, oc2: 15dpf). Oc1, maternally transcribed, decreases at 12hpf (p<0.01), increases after 30dpf (p<0.01). Oc2, detected at 15dpf, increases at 45dpf (p<0.001). Age-size conversion under graphs. Hy1 – Hypural 1; IV – intervertebral space; n.d. – not detected. Scale bars: (**a**-**b**, **g’**-**h’**) 10 μm; (**c**-**f**, **k**) 1mm; (**g**-**j**, **k’**-**k”**) 0.5mm.

In order to clarify possible factors in chordacentrum mineralization, both in “ring” and caudal fin centrum, we have localized Alkaline phosphatase (ALP) and Osteocalcin (Figure 6).

In “ring” centra, ALP-positive signal is co-localized with the site of chordacentrum mineralization prior to any bone deposition outside the notochord sheath. The flattened shape of both outer and inner notochord cells makes it difficult to clearly distinguish the origin of the ALP signal, due to its membrane-bound characteristic (Figure 6a). In caudal fin centra, the arch chondrocytes, particularly those adjacent to the mineralizing notochord sheath, co-localize with a strong ALP signal (Figure 6b).

For Osteocalcin immunostaining, we used an antibody raised against *Argyrosomus regius* Oc1, which was previously validated [36]. It was considered to detect a single protein in zebrafish, since a western blot analysis gave a single band of approximately 5kDa. Nevertheless we cannot guarantee that it distinguishes zebrafish Oc1 from Oc2. However, since Oc2 appears only after day 7 post-fertilization in zebrafish as shown by qPCR analysis (Figure 6l, m), immunostaining in younger fish, such as during formation of chordacentra C1 to C5 (Figure 2a) could only represent sites of Oc1 protein accumulation.

In “ring” (Figure 6c) and caudal fin centra (Figure 6d), Oc1 protein accumulation is observed in the mineralizing matrix of the notochord sheath, even at very early stages of chordacentrum formation. Oc1 accumulation co-localizes with chordacentrum mineralization, reflecting the exact same pattern as observed with alizarin red staining (Figure 3b, b’, c, c’). Moreover, in later stages of centrum formation, we can detect progression fronts in “ring” centra (Figure 6e) while in caudal fin centra we observe homogenous protein accumulation (Figure 6f). In the caudal fin region, Oc1 is also detected in the vertebral arches and caudal fin rays (Figure 6d, f), which are examples of perichondral and intramembranous mineralization, respectively.

Analysis of semi-thin sections of the immunostained samples confirms accumulation of Osteocalcin in the notochord sheath and not around the notochord, during early (Figure 6g, g’, h, h’) and late (Figure 6i, j) stages of chordacentrum formation. In caudal fin centra (Figure 6h, j), the arch chondrocytes adjacent to the mineralizing notochord sheath also co-localize with Oc1 protein (Figure 6h’), as observed for ALP. No perichondral ossification occurred in the arch at sites positive for ALP and Oc1, suggesting that these signals are not associated to arch ossification.

Observation of the transgenic fish *Tg*(*oc2:gfp; osx:mcherry*) showed that no *oc2* expression is present during early stages of centrum formation. In fact, *oc2* is completely absent during chordacentrum formation, appearing first in intramembranous formation of abdominal and caudal arches, a process previously described as mediated by mature osteoblasts (Figure 6k, k’, k”). Analysis of relative *oc1* and *oc2* expression levels during zebrafish development shows that, while *oc1* maternal transcripts are detected in early developmental stages, when no mature osteoblasts can yet be detected (Figure 6l), *oc2* is only expressed after 7dpf (Figure 6m). Chordacentrum formation of the first vertebrae starts at 4.0 mm TL, as previously indicated, which, despite the natural variation in growth, corresponds to fish younger than 7 days.

## Discussion

Zebrafish and medaka vertebral column development and morphology have been extensively described (*e.g.*[1,12,19,29,31,37]). Nevertheless, many questions remain unanswered. In particular, how do distinct mineralization patterns relate to regional differences in arch histogenesis and to susceptibility for vertebral fusion? Why do vertebrae in the caudal fin region fuse as part of the developmental process, in contrast to anterior regions where vertebrae remain individualized [15,30]?

Here, we describe two distinct mineralization patterns of vertebral centra associated with specific vertebral regions. We characterize these patterns based on (1) how mineral is being deposited throughout the centrum, (2) timing of development (prior to or after arch formation) and (3) arch histogenesis (intramembranous or cartilaginous origin). While describing this process we localized accumulation of *oc1* protein in mineralizing notochord sheath, suggesting it could play a function in early mineralization events during chordacentrum formation.

### Notochord segmentation, arch contribution to centra and association with fusion

The first mineralization of the vertebral bodies, independent of the vertebral type (Weberian, abdominal, caudal and caudal fin), occurs within the notochord sheath, establishing the chordacentrum. These are the first indications of segmented units in the zebrafish notochord. Other authors have already described that early centrum mineralization in teleosts occurs within the notochord sheath [2,12,13,19,38,39]. In addition, the notochord may actively contribute to segmentation and centrum formation [2,12,20].

Thus, while the chordacentrum is always established through mineralization of the notochord sheath, the pattern of mineralization differs between caudal fin and remaining vertebral regions: (1) a caudal fin centrum mineralizes after arch formation, while a “ring” centrum is always formed prior to haemal or neural arches, (2) the modified arches from caudal fin vertebrae are composed of cartilage while “ring” centra outside the Weberian apparatus are associated to arches formed by intramembranous ossification; and (3) caudal fin centra display a basiventral origin of mineralization, while all other vertebrae start with a “ring”-shaped mineralization.

Arch histogenesis appears to be a clear mark that distinguishes caudal fin vertebrae from the remaining regions. A similar, regional distinct pattern of mineralization has been observed in medaka, with cartilaginous arches in the caudal fin region and intramembranous arches in the anterior part of the vertebral column [19,40]. Although notochord mineralization of anterior centra in medaka starts dorsal, subsequently chordacentrum mineralization will proceed as “ring” centra [19]. Yet, “ring” notochord-based centrum formation, is not an unique feature of zebrafish and medaka, occurring also in other taxa [41]. Considering caudal fin centra of zebrafish and medaka, both display similar patterns of mineralization [40], linked to the presence of modified cartilaginous arches. Many basal ray-finned fishes have cartilaginous arches directly attached to an unconstricted notochord [18] suggesting that cartilaginous arches are an ancestral feature. Therefore, the caudal fin endoskeleton of zebrafish, and also medaka, appear to display the evolutionarily conserved condition, with cartilaginous arch formation.

Mineralization in the caudal fin centra shows a clear association with attachment sites of the cartilaginous haemal arches to the notochord (basiventral mineralization pattern). The association between basiventral mineralization and presence of cartilaginous haemal arches has also been described in other teleost species, such as the Goldeye (*Hiodon alosoides*) [42], Atlantic salmon [2] and White seabream (*Diplodus sargus*) [43]. In these species too, centrum mineralization originates exactly where the cartilaginous haemal arches are attached to the notochord. Mineralization then progresses from a basiventral origin through bilateral wedge-shaped areas that meet dorsally.

Moreover, we have identified mineralization related proteins in the chondrocytes of the cartilaginous arches, in the immediate vicinity of the area of the incipient mineralization of the notochord. This supports the possible role of the cartilaginous arches in early centrum establishment, a hypothesis that will be discussed in more detail in the next section.

The cartilaginous arches appear to be strictly associated not only with chordacentrum formation but also with the start of autocentrum formation, given that the arches are directly attached to the notochord, affecting bone deposition around the notochord.

**Figure 7:**
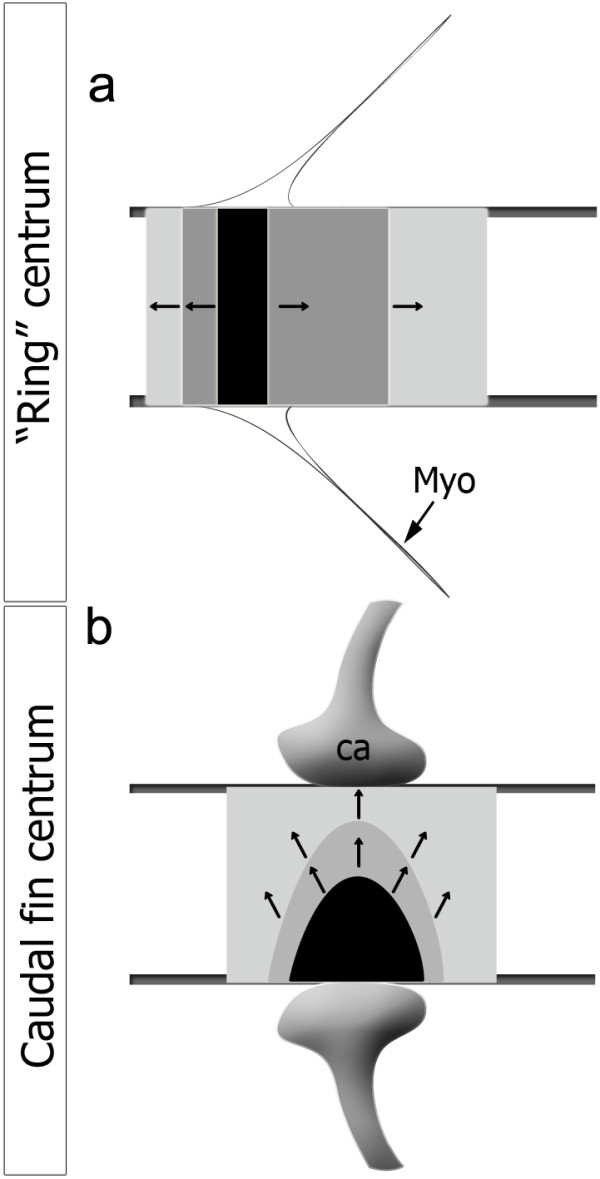
**Progress of mineralization in “ring” and caudal fin centra.** Mineralization expansion is represented by the arrows, in (**a**) a “ring” centrum that expands in linear mineralization fronts in the antero-posterior axis and (**b**) in caudal fin centrum, directly associated with the haemal cartilaginous arch (ca) attached to the notochord sheath, expanding from a basiventral origin. In black is represented the area of initial mineralization, which matches the myoseptum (Myo) in the case of a “ring” centrum.

The type of arches and associated pattern of centrum mineralization appears to be correlated not only to the formation of the vertebral bodies but also to their ability to fuse or stay as individual units (Figure 7). In the medaka *bis* (*biaxial symmetries*) mutant, centra mineralize at sites where ectopic hypurals attach to the notochord, leading to the development of fused centra [40]. Thus, it is tempting to suggest from the zebrafish and medaka data, that the cartilaginous arches have a role in centrum mineralization. Perhaps the predisposition to develop vertebral fusions is related to a specific mineralization pattern with basiventral origin (cartilaginous arch associated).

### Distribution of notochord inner and outer cells during centrum formation

In Atlantic salmon, notochord sheath mineralization is preceded by an apparent segmentation of the chordoblast layer [2]. The same holds for medaka (PEW personal observations). In contrast, differential chordoblast distribution in zebrafish is only clearly observed after chordacentrum mineralization, with few scattered squamous cells in the mineralized area and large, dorso-ventrally oriented, highly proliferative cells in the intervertebral region.

Regarding notochord outer cells, it appears that the segmental pattern of centra in the caudal fin region is associated with the anatomical pattern of arches. In contrast, no association can be found in “ring” centra between outer cell distribution and early chordacentrum formation. The latter is in agreement with the data from zebrafish *fused somite mutants* (*fss*), where paraxial mesoderm lacks a proper segmentation [44]. This mutant shows fusion of vertebral arches, while vertebral centra are individualized, suggesting that centrum segmentation does not rely on sclerotome patterning only. The resegmentation process in zebrafish has been suggested to follow non-lineage restricted compartments, where one somite contributes to more than one vertebral body, independently of the antero-posterior somitic domains (leaky-resegmentation) [1].

Although mineralization is not preceded by a clear pattern of inner or outer cell distribution (exceptions are arch-chondrocytes in the caudal fin), these cells may nevertheless contribute to centrum mineralization. In Atlantic salmon, initial centrum mineralization has been related to chordoblasts, followed by sclerotome-derived bone formation [2,10]. Different from Atlantic salmon, in medaka sclerotome-derived osteoblasts, with no input of chordoblasts, have been described to form vertebral centra [19,35]. Yet, recent data show that conditional ablation of osteoblasts in medaka maintains notochord mineralization and even leads to vertebral fusion [20]. This suggested that notochord cells have the ability to induce mineralization of the notochord sheath. This is consistent with studies in zebrafish [12,45] that show that notochord cell ablation prevents early centrum mineralization.

### Osteocalcin and chordacentrum formation

Osteocalcin is the most abundant noncollagenous protein in bone of vertebrates, including all teleosts analyzed so far [46]. Although Osteocalcin was already described in the vertebral bodies of teleosts including zebrafish [47-50], no previous reports demonstrated Oc in early notochordal mineralization events. Thus, the presence of Oc in stages of chordacentrum mineralization has to be considered in a perspective different from that of regular bone formation.

Within teleosts, Osteocalcin is represented by two distinct isoforms (Oc1 and Oc2) [25,51], encoded by two different genes, which were proposed to originate from a duplication event [25]. Analysis of relative gene expression levels by qPCR for *oc1* and *oc2* showed that *oc2* expression is first detected after 7dpf, while *oc1* is maternally transcribed, suggesting a different role of the two isoforms. In fact, *oc1* is detected prior to any osteoblast differentiation or mineralization event. This leads to the hypothesis that *oc1* must be involved in processes prior to bone formation. Detection of Oc1 by immunostaining shows that this protein accumulates exactly where mineralization of the chordacentrum occurs. In the “ring” centra, the origin of the secreted protein is difficult to assess, due to the squamous morphology of the cells of the notochord epithelium and the inconspicuous presence of cells outside the notochord. In the caudal fin region, ALP positive chondrocytes, directly adjoining the mineralizing notochord sheath, are positive for Oc immunostaining, as well as notochord inner cells. In the *Tg*(*oc2:gfp; osx:mcherry*) fish, *oc2* expression was located at the bone of arches of the “ring” centra, that form via intramembranous bone formation. This time point corresponds to the phase of autocentrum mineralization, as previously described in medaka [52]. Therefore, our results suggest that Oc1, but not Oc2, is connected to chordacentrum mineralization. Likewise, in larval and juvenile Atlantic cod [53], *oc1* and *oc2* are expressed in different cells associated with mineralizing structures. In Atlantic salmon, some mineralized areas with reduced expression of *osteocalcin* (*oc1*) were considered to be associated with expression of a second isoform that the authors did not identify [49]. Laizé and co-workers [25] described Oc2 propeptide as serine-rich, potentially phosphorylated and containing acid residues. These characteristics allow this protein to bind numerous calcium ions. If Osteocalcin has an active role in the process of mineralization or if it is a bone-related hormone that passively binds to calcium [54,55], is a current debate and must be further explored.

## Conclusions

In conclusion, we have analyzed distinct mineralization patterns in different regions of the zebrafish vertebral column and related them to the timing and nature of vertebral arch formation. We have examined the distribution of cells within and outside the notochord, and critically assessed their potential contribution to chordacentrum and autocentrum formation. If notochord mineralization occurs prior to arch formation, the mineralization of the notochord sheath is ring-shaped. If notochord mineralization occurs after cartilaginous arch formation, mineralization of the notochord sheath starts at the insertion point of the arches, with a basiventral origin. Finally, we have detected Oc1 in the notochord sheath during chordacentrum formation, whereas *oc2* was only expressed later in association with bone formation. These results emphasize the need to better characterize the function of both osteocalcin genes in zebrafish and throughout the vertebrate clade.

## Methods

### Ethics statement on animal experiments

Animal handling and experiments were legally accredited by the Portuguese Direcção Geral de Veterinária (DGV) and all the experimental procedures involving animals followed the EU (Directive 86/609/CEE) and National (Directives 1005/92 from October 23, 466/95 from May 17 and 1 1131/97 from November 7) legislation for animal experimentation and welfare.

### Sampling and wholemount skeletal analysis

To establish a timeline of events for early vertebrae development, an ontogenic series of *Danio rerio* was studied. Zebrafish eggs were obtained from natural spawning of wild-type fish and larvae were maintained and reared at standard conditions [56]. Fish were collected at size intervals of 0.1 mm, between 4.0 and 8.0 mm of total length (TL). Similar-sized specimens were always selected among age-matched fish in order to avoid additional interspecific variation.

Larvae were euthanized with a lethal dose of MS222 (Sigma-Aldrich, St. Louis, MO) and fixed for 24 h in 4% buffered paraformaldehyde at 4ºC.

Early mineralization stages of the vertebral bodies were observed following 10 minutes staining with 0.01% alizarin red S in 70% ethanol solution. Specimens were observed under a Zeiss Axio Imager microscope, through fluorescence detection [57].

### Light and transmission electron microscopy (TEM)

Zebrafish specimens of 5.0 and 11.0 mm TL with no pre-staining were fixed in a mixture of 1.5% glutaraldehyde and 1.5% paraformaldehyde in 0.1 M cacodylate buffer and processed for embedding in Epon, according to procedures previously described [58]. Parasagittal 1 μm semi-thin sections of the sites of interest were stained with toluidine blue for 1–2 min (0.2% toluidine blue, 2% Na_2_CO_3_), rinsed with water, air-dried and mounted with DPX (Fluka, Buchs, Switzerland). Ultrathin sections were contrasted with uranyl acetate and lead citrate and observed with a Jeol JEM 1010 (Jeol, Tokyo, Japan), operating at 60 kV. Images were digitized using a DITABIS system (Pforzheim, Germany).

### Identification of sites of mineral deposition and Alkaline phosphatase activity (ALP)

To determine sites of mineral deposition and of Alkaline phosphatase (ALP) activity during centrum formation, specimens ranging from 5.0 to 6.5 mm TL were dehydrated and embedded in glycol methacrylate, as previously described [59], and sectioned at 5 μm.

For detection of minerals, a silver nitrate coupling method, according to von Kossa [60], was used. Sections were counterstained with neutral red. Slides were examined for the appearance of optimal signal, rinsed in distilled water and mounted with DPX (Sigma-Aldrich, St. Louis, MO).

Alkaline phosphatase activity was detected using a previously described method [61] with some modification. Briefly, slides were incubated in staining buffer (100 mM Tris/HCl, pH 9.5; 50mM MgCl_2_; 100 mM NaCl) for 10 min. ALP was demonstrated by adding 4-nitro blue tetrazolium chloride (NBT; 0.38 mg ml^-1^; Roche) and 5-bromo-4-chloro-3-indolyl phosphate (BCIP; 0.175 mg ml^-1^; Roche) to the buffer solution, for a maximum of 30 min at 37ºC, in the dark. Negative control slides were incubated for 10 min at 85ºC in staining buffer prior to addition of NBT and BCIP. Slides were examined for the appearance of optimal signal-noise ratio, counterstained with Nuclear Fast Red Solution (Sigma-Aldrich, St. Louis, MO) and mounted in DPX (Sigma-Aldrich, St. Louis, MO).

### Immunolocalization of PCNA, Osteocalcin and Collagen type II

For detection of cell proliferation through Proliferating cell nuclear antigen (PCNA) immunolocalization, 6.2 mm TL fish were paraffin-embedded according to routine procedures and serially sectioned at 7 μm. A mouse monoclonal primary antibody produced against human antigen (Sigma-Aldrich, St. Louis, MO, dilution 1:1000) was used [62] together with a goat polyclonal anti-mouse immunoglobulin biotin-coupled secondary antibody (Dako, dilution 1:500).

Immunohistochemical detection of Osteocalcin and Collagen type II was performed on fish of 4 to 6.5 mm TL as wholemount staining, according to a procedure previously described [63], using an anti-meagre (*Argyrosomus regius*) Osteocalcin rabbit polyclonal primary antibody, previously validated for zebrafish [36]. For Collagen type II an anti-chicken mouse antibody (II-II6B3, Hybridoma Bank) previously validated for zebrafish [64], was used. Control fish were treated with secondary antibody alone.

Osteocalcin stained specimens were subsequently dehydrated, embedded in epon, serially sectioned at 4 μm and mounted with DPX (Sigma-Aldrich, St. Louis, MO), to achieve a more detailed analysis. Wholemount fish stained for Collagen type II were observed using a Leica MZ Apo stereomicroscope. Sections were observed using a Zeiss Axio Imager Microscope and photographed using an Axiocam MRC videocamera.

### *oc2* transgenic line

The transgenic zebrafish line *Tg*(*oc2:gfp; osx:mcherry*), previously described by [65], was provided by Stefan Schulte-Merker from Hubrecht Institute-KNAW and University Medical Centre (Uppsalalaan 8, 3584 CT Utrecht, The Netherlands).

### RNA extraction and *oc1*/*oc2* quantitative expression analysis

Total RNA was extracted from a pool of up to twenty specimens following the Chomczynski and Sacchi method [66]. Samples were collected at stages 8-32 or 128 cells, at shield stage, at 12, 24, 48, 72 and 96 hours post-fertilization (hpf) and at 5, 7, 15, 30, 45 and 60 days post-fertilization (dpf). To prevent genomic DNA contamination, following RNA extraction, samples were DNase treated with RQ1 DNase (Promega, Madison, WI) according to the manufacturer’s protocol, followed by a phenol-chlorophorm purification step. RNA quantity and quality was determined by spectrophotometry (NanoDrop ND-1000, Thermo Scientific) and electrophoresis.

cDNA was prepared from 1μg of total RNA from each sample using Moloney-murine leukemia virus (M-MLV) reverse transcriptase (Invitrogen), RNase Out (Invitrogen), and an oligo(dT) adapter (5'-ACGCGTCGACCTCGAGATCGATG(T)13-3') in a total volume of 20μl for 1h at 37ºC. For quantitative real-time PCR a StepOnePlus 96 (Applied Biosystems) was used together with 1× SsoFastTM EvaGreen Supermix (BioRad, Richmond, USA), 0.2 μM of forward (Oc1: 5^′^-GAAGCGAACATGAAGAGTCTGACAGTCC-3^′^; Oc2: 5^′^-CCAACTCCGCATCAGACTCCGCATCA-3^′^) and reverse (Oc1- 5^′^-TTTATAGGCGGCGATGATTCC-3^′^; Oc2: 5^′^-AGCAACACTCCGCTTCAGCAGCACAT-3^′^) primers and 200 ng of reverse-transcribed RNA. PCR conditions were 15 min at 95°C followed by 40–50 amplification cycles (each cycle is 30 s at 95°C, 15 s at 68°C). Expression values were normalized using the *Elongation factor 1α (Ef1α)* housekeeping gene [67]. Gel electrophoresis and melt curve analysis were used to confirm specific product formation.

All data were statistically analyzed using a non-parametric Kruskal-Wallis test, followed by Wilcoxon multiple comparison with the GraphPad Prism software.

### Terminology

The subdivision of the vertebral column in different regions is based on the nomenclatures of Arratia et al. [16], Bird and Mabee [31] and Nybelin [68] (Figure 1). From anterior to posterior, vertebrae were identified as (1) Weberian vertebrae, (2) abdominal vertebrae (rib-bearing with open haemal arches and without haemal spines), (3) caudal vertebrae (with haemal arches closed), and (4) caudal fin vertebrae (with modified haemal and neural arches and respective spines). The nomenclature of the Weberian apparatus follows [31]. The caudal fin vertebrae nomenclature follows Patterson [69], adapted by Arratia and Schultze [70] and Bensimon-Brito et al. [32]. The caudal fin vertebral region contains preural and ural vertebral bodies. Preural vertebral bodies (abbreviated as PU) have haemal and neural arches that support the caudal fin. Ural vertebral bodies (briefly urals, abbreviated as U) support the caudal fin with modified haemal arches that do not enclose the caudal artery. A plus sign is added in superscript when a vertebral centrum is derived from a fusion event (compound centrum), as described previously [32].

## Competing interests

The funding agencies had no role in study design, data collection and analysis, decision to publish, or preparation of the manuscript. The authors have declared no competing interests.

## Authors’ contributions

Conceived and designed the experiments: ABB, MLC, AH, PEW. Performed the experiments: ABB and JC. Analyzed the data: ABB, JC, MLC, AH, PEW. Contributed reagents/materials/analysis tools: MLC, AH. PEW. Contributed to the writing of the paper: ABB, MLC, AH, PEW. All authors read and approved the final manuscript.
